# Expression of EZH2 in uveal melanomas patients and associations with prognosis

**DOI:** 10.18632/oncotarget.19462

**Published:** 2017-07-22

**Authors:** Ying Cheng, Ying Li, Xin Huang, Wenbin Wei, Yi Qu

**Affiliations:** ^1^ Department of Geriatrics, Qilu Hospital of Shandong University, Jinan, China; ^2^ Beijing Tongren Hospital, Capital Medical University, Beijing Tongren Eye Center, Beijing Ophthalmology and Visual Science Key Laboratory, Beijing, China

**Keywords:** uveal melanomas, EZH2, ki-67, immunohistochemistry, prognosis

## Abstract

**Purpose:**

To analyze the prognostic value and potential target for therapeutic intervention of enhancer of zeste homologue 2 (EZH2) in uveal melanomas (UM) patients.

**Method:**

We analyzed EZH2 expression in 89 primary UM patients by immuno- histochemistry to observe the clinicopathological and prognostic value of EZH2.

**Results:**

The high levels of mitoses count and Ki67 labeling index had significant correlation with overexpression EZH2 (R = 0.408, P

**Conclusion:**

Our critical finding is that overexpression EZH2 in UM can be served as predictive marker and is associated with adverse clinical outcomes. Further observation of EZH2 as a potential therapeutic target in UM is necessary.

## INTRODUCTION

Uveal melanoma (UM) is the most common primary intraocular malignant tumor in adults. UM eventually spread to the liver in up to 50% of patients and nearly half of the patients have subclinical metastasis at the time of diagnosis [1, 2]. There are several treatment measures on UM in clinic nowadays, the main alternatives include enucleation, proton beam radiotherapy and plaque radiotherapy. Although the progress and availability of alternative therapeutic models, the survival rates of UM patients are nearly unchanged in 30 years [3]. Once metastasis occurs, median survival is only 6 month with or without treatment [4].

The increased risk of developing metastatic disease in UM was reported to be associated with clinic and morphological factors such as larger tumor size, presence of epithelioid cells, closed vascular loops, mitotic activity, nodular growth and extracellular matrix patterns [5–8]. Molecular studies have also shown that cluster differentiation could be made, classifying tumors according to their low and high risk of metastasis. In our previous study, the poor outcome of UM was related with overexpression of high mobility group A1 protein (HMGA1) [9]. Genetic studies reported the loss of chromosome 3 was the risk factor of poor outcome of UM [10, 11]. However, none of the above indicators can be considered as effectively therapeutic targets in UM except HMGA1. Therefore, it is worth identifying reliable biomarkers of uveal melanoma for its early diagnosis and effective therapy.

Enhancer of zeste homologue 2 (EZH2) is a member of the polycomb group of genes, regulating the cell cycle through nucleosome modification, chromatin remodeling, and interacting with other transcription factors [12], the expression of EZH2 delays upon tissue maturation and differentiation [13]. EZH2 overexpression has been reported to be related with increased tumor cell proliferation and worse outcome in several carcinomas including breast cancer [14], endometrial carcinomas [15] and hormone-refractory prostate cancer [16], which indicated that the expression of EZH2 protein might serve as the potential biomarker in carcinomas. To the best of our knowledge, no report was published on the role of EZH2 protein in UM prognosis.

In the present study, a series of UM cases were analyzed the prognostic value and potential target for therapeutic intervention of EZH2 by using immunohistochemistry. The correlation of EZH2 protein with cell proliferation marker Ki67/MIB-1 and relevant clinical parameters were explored herein.

## RESULTS

### Baseline characteristics

This study involved 89 UM patients in which 49(55%) were males and 40 were females. The mean age of the subjects was 46±14.5 years. According to American Joint Committee on Cancer (AJCC) 7th criteria, tumor categories were stage I in 5 (6%)patients, stage IIA in 24 (27%)patients, stage IIB in 26(29%) patients, stage IIIA in 24(27%) patients, stage IIIB in 7 (8%) patients and stage IIIC in 3 (3%) patients. The average follow-up time of 89 patients was 78.1±26.4 months (median =81 months; range: 8–144 months). Other details of patients’ parameters listed in Table 1 have been described earlier [9].

**Table 1:** Clinical, pathologic characteristics according to EZH2 alterations in uveal melanoma

		EZH2 nulear expression		
Clinical, pathologic features	Total N	High	Low	P value
	89	31(35%)	58(65%)	
**Gender**				0.26
**Male,n(%)**	49(55%)	20(65%)	29(50%)	
**Female,n(%)**	40(45%)	11(35%)	29(50%)	
**Mean age at diagnosis** **± SD**	46.0±14.5	46.1±14.1	45.3±15.1	0.19
**Laterality**				
**Left eye, n(%)**	38(43%)	14(45%)	24(41%)	0.53
**Right eye, n(%)**	51(57%)	17(55%)	34(59%)	
**Largest basal tumor diameter (mm)**				
**Mean (range)**	14.0(7-21)	14.5(11-18)	13.9(9-21)	0.10
**<15, n(%)**	59(66%)	20(65%)	39(67%)	
**>15, n(%)**	30(34%)	11(35%)	19(63%)	
**Tumor thickness(mm)**				0.63
**Mean (range)**	10.1(3-20)	10.2(3-20)	9.6(5-16)	
**<10, n(%)**	46(52%)	16(52%)	30(51%)	
**>10, n(%)**	43(48%)	15(48%)	28(49%)	
**Tumor growth pattern (Nodular)**				0.23
**Yes, n(%)**	42(47%)	16(52%)	26(45%)	
**No, n(%)**	47(53%)	15(48%)	32(55%)	
**Cillary body involvement**				0.64
**Yes, n(%)**	17(19%)	8(26%)	9(15%)	
**No, n(%)**	72(81%)	23(74%)	49(85%)	
**Optic disc involvement**				0.43
**Yes, n(%)**	9(10%)	6(19%)	3(6%)	
**No, n(%)**	80(90%)	25(81%)	55(94%)	
**AJCC classification**				
**Stage I** (**T1a)**	5(6%)	1(4%)	4(7%)	
**Stage IIA (T1b-d and T2a)**	24(27%)	7(22%)	17(29%)	
**Stage IIB (T2b and T3a)**	26(29%)	10(32%)	16(28%)	
**Stage IIIA (T2c-d, T3b-c and T4a)**	24(27%)	9(29%)	15(26%)	
**Stage IIIB (T3b and T4b-c)**	7(8%)	3(9%)	4(7%)	
**Stage IIIC (T4d-e)**	3(3%)	1(4%)	2(3%)	
**Stage IV (Any T N1/M1)**	0(0%)	0(0%)	0(0%)	
**Closed loop**				0.51
**Yes, n(%)**	29(33%)	6(19%)	23(39%)	
**No, n(%)**	60(67%)	25(81%)	35(61%)	
**Extraocular spread**				0.64
**Yes, n(%)**	11(12%)	7(23%)	4(6%)	
**No, n(%)**	78(88%)	24(77%)	54(94%)	
**Epithelioid cells**				0.012
**Yes, n(%)**	13(15%)	10(32%)	3(5%)	
**No, n(%)**	76(85%)	21(68%)	55(95%)	
**Mitoses count/ 40 HPF**				<0.0001
**≤4, n(%)**	69 (76%)	14(45%)	55(95%)	
**>4, n(%)**	20 (24%)	17(55%)	3(5%)	
**Ki67 labeling index**				<0.0001
**≤2, n(%)**	70(79%)	19(61%)	51(88%)	
**>2, n(%)**	19(21%)	12(39%)	7(12%)	
**Follow-Up Time (Years)**				0.31
**Mean** **± SD**	78.1±26.4	76.7±26.3	78.4±26.2	
**≤1**	16(18%)	9(29%)	7(12%)	
**>1 and ≤3**	23(26%)	11(35%)	12(21%)	
**>3 and ≤5**	24(27%)	7(23%)	17(29%)	
**>5 and ≤10**	25(28%)	4(13%)	21(36%)	
**>10**	1(1%)	0(0%)	1(2%)	

AJCC, American Joint Committee on Cancer

### Correlation between EZH2 expression and other characteristics in UM patients

**Figure 1:**
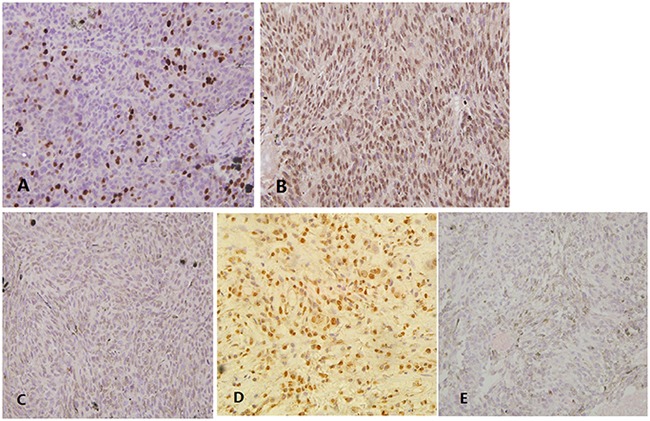
Expression of EZH2 in UM and positive/negative controls **(A)** Detection of enhancer of zeste homologue 2 (EZH2) immunoreactivity in UM. **(B)** High level of EZH2 expression in UM. **(C)** Negative case without nuclear staining. **(D)** Using squamous cell carcinomas as positive controls. **(E)** No primary antibody served as negative controls.

Detection of EZH2 immunoreactivity in UM was shown in Figure 1A with clear nuclear staining. High level of expression, negative case without nuclear staining, positive control and negative control were shown in Figure 1B-1E, respectively. EZH2 nuclear expression was detected in 51 UM samples (57%), 31(35%) expressed high levels among them (Table 1).

**Figure 2:**
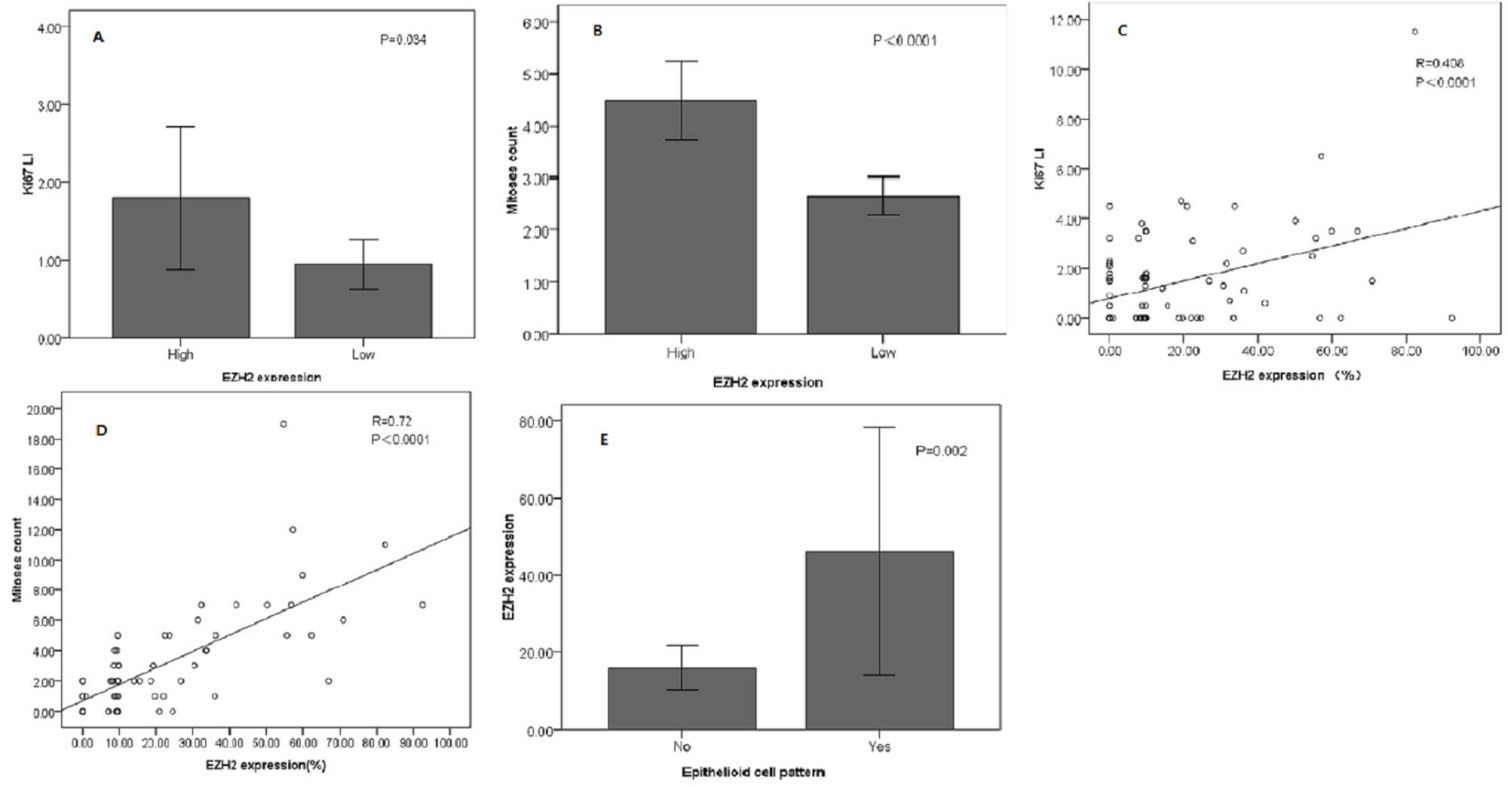
Correlation between EZH2 expression and other parameters in UM The Ki67 LI **(A)** and mitoses counts **(B)** were significantly higher in high EZH2 expression group than in low EZH2 expression group. Significant correlations have been found between the expression of EZH2 and Ki67 LI **(C)**, and mitoses counts **(D)**. EZH2 expression levels were significantly higher in cases showing epithelioid cell pattern than in cases lacking this cell pattern **(E)**.

The presence of epithelioid cells, high level of mitoses count and high Ki67 LI had significant correlations with overexpression EZH2 (P=0.012,<0.0001,<0.0001, Table 1). Additionally, Ki67 LI and mitoses count were represented significantly higher in high EZH2 expression group than low expression group (P = 0.034,<0.0001) (Figure 2A, 2B). The above correlations were confirmed again by Pearson's correlation coefficient analysis (R = 0.408, P<0.0001; R = 0.72, P<0.0001) (Figure 2C, 2D). Furthermore, we also found that the EZH2 expression was also significantly higher in epithelioid cell pattern of UM (P = 0.002; Figure 2E). However, no significant correlations were found between EZH2 expression and other parameters including gender, basal tumor diameter and tumor thickness etc. (Table 1).

### EZH2 impact on UM patient survival

**Figure 3:**
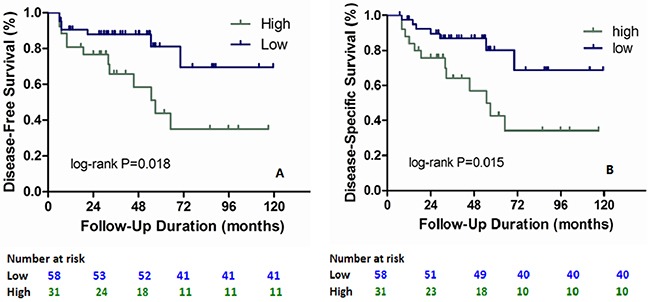
Kaplan-Meier curves for survival in UM patients Survival of UM patients were evaluated according to EZH2 expression and Ki67 LI. Significant differences in disease-free survival rates were observed **(A)**. Significant differences in disease-specific survival rates were also observed **(B)**. P values were calculated with a log-rank test.

Figure 3A shows Kaplan–Meier curves for the impact of high EZH2 expression was significantly associated with high trend of distant metastases during disease-free survival period (log rank P = 0.018). Moreover, significant associations were found between presence of epithelioid cell pattern (hazard ratio (HR), 3.90; P = 0.021), high mitoses count (HR, 1.40; P = 0.0001), high level of Ki67 LI (HR, 1.23; P = 0.012), overexpression of EZH2 (HR, 3.64; P = 0.035) and higher risk of metastases by univariate Cox regression (Table 2). When using multivariate Cox regression, we found high mitoses count (HR, 1.39; P = 0.0001), high level of Ki67 LI (HR, 1.64; P = 0.019) and overexpression of EZH2 (HR, 2.12; P = 0.037) were significantly related to increased risk of metastases (Table 2).

**Table 2:** Clinicopathological features, tumor markers, EZH2, and uveal melanoma patients’ survival

Total N (%)			No.of events	Univariate HR(95% CI)	*P*	Multivariate HR^1^ (95% CI)	*P*
Disease-free survival							
Epithelioid cells	No	76(85%)	25	1(reference)		1(reference)	
	Yes	13(15%)	6	3.90(1.23 to 12.33)	0.021	2.22(0.64 to 7.70)	0.208
Mitoses count	≤4	69(76%)	14	1(reference)		1(reference)	
	>4	20(24%)	17	1.40(1.23 to 1.59)	0.0001	1.39(1.19 to 1.62)	0.0001
Ki67 LI	≤2	70(79%)	18	1(reference)		1(reference)	
	>2	19(21%)	13	1.23(1.05 to 1.45)	0.012	1.64(1.09 to 2.46)	0.019
EZH2	Low	58(65%)	20	1(reference)		1(reference)	
	High	31(35%)	11	3.64(1.09 to 8.11)	0.035	2.12(0.51 to 8.85)	0.037
Disease-specific survival							
Epithelioid cells	No	76(85%)	25	1(reference)		1(reference)	
	Yes	13(15%)	6	3.62(1.24 to 11.46)	0.029	2.00(0.59 to 6.78)	0.267
Mitoses count	≤4	69(76%)	14	1(reference)		1(reference)	
	>4	20(24%)	17	1.43(1.25 to 1.64)	0.0001	1.39(1.20 to 1.62)	0.0001
Ki67 LI	≤2	70(79%)	18	1(reference)		1(reference)	
	>2	19(21%)	13	1.22(1.04 to 1.44)	0.017	1.45(1.02 to 2.08)	0.041
EZH2	Low	58(65%)	20	1(reference)		1(reference)	
	High	31(35%)	11	5.17(1.42 to 8.80)	0.013	3.92(1.44 to 7.85)	0.036

HR, hazard ratio; CI, confidence interval.

^1^The multivariate Cox regression model initially included the EZH2 expression variable (high or low), age of diagnosis, sex, largest basal tumor diameter, tumor thickness and epithelioid cell pattern.

### EZH2 impact on melanoma-specific survival of UM patient

During disease-specific survival, Kaplan–Meier curves showed overexpression of EZH2 was significantly associated with high risk of metastasis in Figure 3B (log rank P = 0.015). In addition, presence of epithelioid cell pattern (HR, 3.62; P = 0.029), high mitoses count (HR, 1.43; P = 0.0001), high level of Ki67 LI (HR, 1.22; P = 0.017) and high EZH2 expression (HR, 5.17; P = 0.013) were found to be significantly related with increased trend of disease-specific mortality using univariate Cox regression (Table 2). And by multivariate Cox regression, we found high mitoses count (HR, 1.39; P = 0.0001), Ki67 LI (HR, 1.45; P = 0.041) and EZH2 expression (HR, 3.92; P = 0.036) were significantly associated with worse prognosis (Table 2).

## DISCUSSION

In this study, we used immunohistochemistry to analyze the EZH2 expression in UM patients and evaluate its prognostic value and potential therapeutic target. Our critical finding is that overexpression of EZH2, associated with presence of epithelioid cell, high level of mitoses counts and Ki67 LI, is related with poor clinical outcomes in UM patients.

Common tumor treatment alternatives including enucleation and plaque brachytherapy can be helpful for preserving the effected eye and vision in some patients. However, UM patient survival has been nearly no change in three decades despite the progress of therapy methods [3]. Hence, an effective candidate is necessary in clinic. EZH2 is a cell cycle regulator played an important role in G2-M transition and E2F regulation [17]. Overexpression of EZH2 has been reported in many malignancies such as breast cancer [14] and B Cell Lymphomas [18]. High EZH2 expression was related to aggressive clinical behavior and poor outcome in malignancy. Recently, three EZH2 inhibitors have been observed in clinical trials including CPI-1205 in B-Cell Lymphomas, E7438 in Advanced Solid Tumors/B Cell Lymphomas and GSK2816126 in Relapsed/ Refractory Diffuse Large B Cell and Transformed Follicular Lymphomas [19].

EZH2 overexpression was found to be linked to shorter survival time of UM patients in this study, consistent with previous studies in other malignancies [20, 21]. In addition, high EZH2 expression and metastatic mortality were associated with other UM parameters such as presence of epithelioid cell pattern, high mitoses count and Ki67 LI. This finding confirmed the role of EZH2 as a reliable marker for prognosis in UM.

Tumor cell pattern is also a helpful prognostic factor for survival time in UM. UM cellular morphology was divided into spindle cell and mixed cell types according to Modified Callender system [22]. McLean IW et al. have reported that the spindle cell type had the best prognosis, mixed cell type a worse prognosis [22, 23]. In this study, we classified the tumor cell type as mentioned above and found the similar correlation in univariate logistic regression analysis. However, when adjusted for other compounding factors, epithelioid cell pattern was no longer significantly associated with survival. It may can be explained that tumor cell type is not play a decisive role in prognostic.

In our patient cohort, clinic features were not significantly associated with the survival rate except mitoses counts. Correspondingly, the conclusions of different studied were various [24–27]. The parameters used as predictive markers may not be the most reliable method evidently. Currently, several studies have reported that one of EZH2 functions is involved in target gene activation and high EZH2 expression is related with many signaling pathways, such as the pRB-E2F, estrogen receptor and c-Myc signal transduction pathways [17, 28, 29]. These results collectively, suggest that the regulation of EZH2 expression is rather complicated, it may serve as a promising marker for UM treatment and can help doctors to judge the tumor prognosis.

In conclusion, we assessed the prognostic value of EZH2 expression and its correlation with clinical and histopathologic parameters by immunohistochemistry. We found that overexpression EZH2 in UM is associated with adverse clinical outcome, hence, it might able to play a part in therapeutic target for reducing the tumor metastasis and improving survival time of UM patients.

## MATERIALS AND METHODS

### Patients and tumor samples

Eighty-nine primary UM samples were collected after surgical enucleation from 1998 to 2006. Full ophthalmologic and systemic clinical examinations, such as liver function tests, chest X-ray and abdominal ultrasonography, were performed regularly before and after operation. Computed tomography or magnetic resonance imaging was used to confirm the possibility of metastasis in other parts of the body. This study conformed to the Declaration of Helsinki and was approved by the ethics committee of the appropriate institutes. All the patients had given their informed consent for experimental research previously.

All the patients were diagnosed at ocular oncology clinic of Qilu hospital or Beijing Tongren hospital and the enucleation was conducted for UM during the indicated period. The duration of the follow-up period began with the date of UM diagnosis to the date of death or last follow-up (January 1^st^ 2016), whichever came first. Eighty-nine patients with complete follow-up data have been examined in survival analyses.

### Histopathologic examination

As our previous study [9], the diagnosis of melanoma was confirmed using sample stained for hematoxylin and eosin (H&E) and/or MelanA. The tumors were histologically examined for cell type, localization, size, mitotic count, necrosis, and scleral invasion. Spindle and mixed cell types were assessed using the modified Callender system [22]. Extravascular matrix patterns were assessed using the periodic acid-Schiff reagent without hematoxylin counterstaining, and the sections were viewed under a green filter [30]. The mitotic count was measured by counting the number of mitoses in 40 high-power fields (HPF) in the H&E sections [31]. UM size, node, and metastasis (TNM) was classified according to the AJCC 7th edition system criteria [32].

### Immunohistochemistry

Histological sections of formalin-fixed paraffin-embedded samples were analyzed for the presence of Ki67 and EZH2 by the labeled streptavidin-biotin method. After deparaffinization and antigen retrieval using an autoclave oven technique, sections were incubated at 4°C overnight and incubated with EZH2 Rabbit Polyclonal Antibody (clone ZMD.309) (1:100; #187395, Thermo Fisher Scientific, Waltham, MA USA 02451) and Ki67 antigen mouse monoclonal antibody (1:75, DakoCytomatin, Glostrup, Denmark) at 4°C. Antigen-antibody complexes were detected by the cobalt-3, 3’-diaminobenzidine reaction. Squamous cell carcinomas known to be positive for EZH2 expression were used as positive controls [33]. Sections incubated in phosphate-buffered saline without the primary antibody served as negative controls.

Images of several HPF (×400) were captured from regions with different staining intensities, including high, moderate, low, and negative staining for each case. The photographs were printed on plain paper, and a grid was drawn over them. A total of 1000 cells were counted and expressed as a percentage of tumor cells with positive nuclei. The percentage of EZH2 positive tumor cells was scored on a scale from 0 to 4 (0, no staining; 1+, ≤10%; 2+,≤30%; 3+, ≤50%; 4+, >50%). The expression levels of EZH2 were divided into two groups according to score: low (score: 0, 1+); high (score: 2+, 3+, and 4+) [9, 34]. The Ki67 labeling index (LI) was determined by counting the number of positive cells in a total of 800–1000 tumor cells observed in regions of highest staining (hot spot) at several HPF(×400). The results were expressed as a percentage of tumor cells with positive nuclei.

### Statistical analysis

Statistical evaluation was performed using SPSS software (IBM SPSS Statistics 21; SPSS Inc., Chicago, IL). All data were described as mean ± standard deviation (SD) if applicable. The Pearson x^2^ test or Fisher's exact test was used to compare qualitative variables. Associations and differences among the different parameters were analyzed using the Mann-Whitney U test and x^2^ test. The relation between the expression levels of EZH2 and Ki67 LI were performed by Pearson's correlation coefficient. The Kaplan-Meier method and log-rank test were used for survival analyses. Cox proportional hazards regression model was used to calculate mortality hazard ratios and 95% confidence intervals (CIs). To control for confounding variables, we used multivariate Cox proportional hazards regression models. To assess independent association between EZH2 expression and key severity markers (epithelioid cells, mitosis count and Ki67 LI), multivariate logistic regression analysis was done and odds ratio (OR) was adjusted for age and gender. Probability values (P)<0.05 were considered to be statistically significant.

## Abbreviations

UM: uveal melanoma
HMGA1: high mobility group A1 protein
EZH2: enhancer of zeste homologue 2
HR: hazard ratio
H&E: hematoxylin and eosin
HPF: high-power fields
AJCC: American Joint Committee on Cancer
SD: standard deviation
CIs: confidence intervals
OR: odds ratio

## References

[1] Bakalian S, Marshall JC, Logan P, Faingold D, Maloney S, Di Cesare S, Martins C, Fernandes BF, Burnier MN (2008). Molecular pathways mediating liver metastasis in patients with uveal melanoma. Clin Cancer Res.

[2] Jager MJ, Dogrusoz M, Woodman SE (2017). Uveal melanoma: identifying immunological and chemotherapeutic targets to treat metastases. Asia Pac J Ophthalmol (Phila).

[3] Papastefanou VP, Cohen VM (2011). Uveal melanoma. J Skin Cancer.

[4] Bedikian AY (2006). Metastatic uveal melanoma therapy: current options. Int Ophthalmol Clin.

[5] Coupland SE, Campbell I, Damato B (2008). Routes of extraocular extension of uveal melanoma: risk factors and influence on survival probability. Ophthalmology.

[6] Damato B, Coupland SE (2009). A reappraisal of the significance of largest basal diameter of posterior uveal melanoma. Eye (Lond).

[7] Folberg R, Pe’er J, Gruman LM, Woolson RF, Jeng G, Montague PR, Moninger TO, Yi H, Moore KC (1992). The morphologic characteristics of tumor blood vessels as a marker of tumor progression in primary human uveal melanoma: a matched case-control study. Human Pathol.

[8] Collaborative Ocular Melanoma Study Group (2001). Assessment of metastatic disease status at death in 435 patients with large choroidal melanoma in the Collaborative Ocular Melanoma Study (COMS): COMS report no. 15. Arch Ophthalmol.

[9] Qu Y, Wang Y, Ma J, Zhang Y, Meng N, Li H, Wang Y, Wei W (2013). Overexpression of high mobility group A1 protein in human uveal melanomas: implication for prognosis. PLoS One.

[10] Prescher G, Bornfeld N, Hirche H, Horsthemke B, Jockel KH, Becher R (1996). Prognostic implications of monosomy 3 in uveal melanoma. Lancet (Lond).

[11] Trolet J, Hupe P, Huon I, Lebigot I, Decraene C, Delattre O, Sastre-Garau X, Saule S, Thiery JP, Plancher C, Asselain B, Desjardins L, Mariani P (2009). Genomic profiling and identification of high-risk uveal melanoma by array CGH analysis of primary tumors and liver metastases. Invest Ophthalmol Vis Sci.

[12] Hussein YR, Sood AK, Bandyopadhyay S, Albashiti B, Semaan A, Nahleh Z, Roh J, Han HD, Lopez-Berestein G, Ali-Fehmi R (2012). Clinical and biological relevance of enhancer of zeste homolog 2 in triple-negative breast cancer. Human Pathol.

[13] Lee TI, Jenner RG, Boyer LA, Guenther MG, Levine SS, Kumar RM, Chevalier B, Johnstone SE, Cole MF, Isono K, Koseki H, Fuchikami T, Abe K (2006). Control of developmental regulators by Polycomb in human embryonic stem cells. Cell.

[14] Collett K, Eide GE, Arnes J, Stefansson IM, Eide J, Braaten A, Aas T, Otte AP, Akslen LA (2006). Expression of enhancer of zeste homologue 2 is significantly associated with increased tumor cell proliferation and is a marker of aggressive breast cancer. Clin Cancer Res.

[15] Bachmann IM, Halvorsen OJ, Collett K, Stefansson IM, Straume O, Haukaas SA, Salvesen HB, Otte AP, Akslen LA (2006). EZH2 expression is associated with high proliferation rate and aggressive tumor subgroups in cutaneous melanoma and cancers of the endometrium, prostate, and breast. J Clin Oncol.

[16] Bryant RJ, Cross NA, Eaton CL, Hamdy FC, Cunliffe VT (2007). EZH2 promotes proliferation and invasiveness of prostate cancer cells. Prostate.

[17] Bracken AP, Pasini D, Capra M, Prosperini E, Colli E, Helin K (2003). EZH2 is downstream of the pRB-E2F pathway, essential for proliferation and amplified in cancer. EMBO J.

[18] Tian X, Pelton A, Shahsafaei A, Dorfman DM (2016). Differential expression of enhancer of zeste homolog 2 (EZH2) protein in small cell and aggressive B-cell non-Hodgkin lymphomas and differential regulation of EZH2 expression by p-ERK1/2 and MYC in aggressive B-cell lymphomas. Mod Pathol.

[19] Marchesi I, Bagella L (2016). Targeting Enhancer of Zeste Homolog 2 as a promising strategy for cancer treatment. World J Clin Oncol.

[20] Scholzen T, Gerdes J (2000). The Ki-67 protein: from the known and the unknown. J Cell Physiol.

[21] Simon JA, Tamkun JW (2002). Programming off and on states in chromatin: mechanisms of Polycomb and trithorax group complexes. Curr Opin Genet Dev.

[22] McLean IW, Foster WD, Zimmerman LE, Gamel JW (1983). Modifications of Callender's classification of uveal melanoma at the Armed Forces Institute of Pathology. Am J Ophthalmol.

[23] Seddon JM, Polivogianis L, Hsieh CC, Albert DM, Gamel JW, Gragoudas ES (1987). Death from uveal melanoma. Number of epithelioid cells and inverse SD of nucleolar area as prognostic factors. Arch Ophthalmol.

[24] Collaborative Ocular Melanoma Study Group: The COMS randomized trial of iodine 125 brachytherapy for choroidal melanoma: V. Twelve-year mortality rates and prognostic factors: COMS report No. 28 (2006). Arch Ophthalmol.

[25] Rietschel P, Panageas KS, Hanlon C, Patel A, Abramson DH, Chapman PB (2005). Variates of survival in metastatic uveal melanoma. J Clin Oncol.

[26] Zloto O, Pe’er J, Frenkel S (2013). Gender differences in clinical presentation and prognosis of uveal melanoma. Invest Ophthalmol Vis Sci.

[27] Barr CC, McLean IW, Zimmerman LE (1981). Uveal melanoma in children and adolescents. Arch Ophthalmol.

[28] Tang X, Milyavsky M, Shats I, Erez N, Goldfinger N, Rotter V (2004). Activated p53 suppresses the histone methyltransferase EZH2 gene. Oncogene.

[29] Tan JZ, Yan Y, Wang XX, Jiang Y, Xu HE (2014). EZH2: biology, disease, and structure-based drug discovery. Acta Pharmacol Sin.

[30] Folberg R, Rummelt V, Parys-Van Ginderdeuren R, Hwang T, Woolson RF, Pe’er J, Gruman LM (1993). The prognostic value of tumor blood vessel morphology in primary uveal melanoma. Ophthalmology.

[31] Damato B, Duke C, Coupland SE, Hiscott P, Smith PA, Campbell I, Douglas A, Howard P (2007). Cytogenetics of uveal melanoma: a 7-year clinical experience. Ophthalmology.

[32] Shields CL, Kaliki S, Furuta M, Fulco E, Alarcon C, Shields JA (2015). American joint committee on cancer classification of uveal melanoma (anatomic stage) predicts prognosis in 7,731 patients: the 2013 zimmerman lecture. Ophthalmology.

[33] Liu F, Gu L, Cao Y, Fan X, Zhang F, Sang M (2016). Aberrant overexpression of EZH2 and H3K27me3 serves as poor prognostic biomarker for esophageal squamous cell carcinoma patients. Biomarkers.

[34] Wang EL, Qian ZR, Rahman MM, Yoshimoto K, Yamada S, Kudo E, Sano T (2010). Increased expression of HMGA1 correlates with tumour invasiveness and proliferation in human pituitary adenomas. Histopathology.

